# Dementia and Adherence to Anti-Diabetic Medications: A Meta-Analysis

**DOI:** 10.7759/cureus.14611

**Published:** 2021-04-21

**Authors:** Hyder Mirghani, Samar Aljohani, Afaf Albalawi

**Affiliations:** 1 Internal Medicine, University of Tabuk, Tabuk, SAU; 2 Family Medicine, University of Tabuk, Tabuk, SAU

**Keywords:** diabetes mellitus, dementia, medications adherence

## Abstract

Introduction

Diabetes mellitus (DM) and dementia (DN) are common morbid disorders with high mortality, the two disorders shared the pathogenesis of proinflammation and insulin resistance. Polypharmacy is expected when DM and DN co-exist and medication adherence is essential to an effective self-care and management plan. This meta-analysis aimed to assess medication persistence among patients with diabetes and cognitive impairment (CogImp).

Methods

We systematically searched the literature through PubMed, Medline, Cochrane library, and the first 100 articles published in Google Scholar. We included articles publishes in English and conducted on humans, no limitation was set to the date of publication, all the articles were approached from the first published up to March 15, 2021. The keywords used were Dementia, cognitive impairment, cognitive decline, cognitive dysfunction, diabetes self-care, compliance to anti-diabetic drugs, and medication adherence. One hundred-seventy-six were identified, the 12 full texts screened, only four fulfilled the inclusion and exclusion criteria.

Results

The studies were published in Europe, the United States, and Asia (all were observational). The results showed no effects of dementia on medication adherence, P-value of 0.41, odd ratio: 1.09, 95% CI: 0.89-1.32, Chi-square for heterogeneity: 12.15, I^2 ^= 75%, and standard difference = 3. The P-value for heterogeneity was 0.007. The studies included 2,556 patients and 1,854 events.

Conclusion

No association was found between dementia and compliance to anti-diabetic medications. Further prospective studies are needed to solve the issue.

## Introduction

Diabetes mellitus (DM) is approaching an epidemic, the disease is of great concern globally due to its increasing prevalence, currently, 9.3% are suffering from the disease, and the suffering is reflected on the families, healthcare system, and national economies.

The number of patients with the diagnosis of DM is expected to grow to 700 million by the year 2045. Besides, 50.1% of people are living with DM but they are not aware of the diagnosis. The direct global expenditure on DM is 760 billion US Dollars in the year 2019 and the projection is 845 for the year 2045 [1, 2]. Dementia (DN) (a seriously disabling disease) is on the rise globally and due to the aging population, it is estimated that 130 million were affected in the year 2015 and this number might not reflect the real size of the problem due to the insufficient data and the challenge of case ascertainment [3].

The growing high body mass index (due to work involving physical inactivity and an unfriendly diet) is mirrored by the emergence of multi-morbid non-communicable diseases (a real healthcare system nightmare). The matter is further complicated by the higher rates of these morbidities in low/middle-income countries lacking well-established care [4]. Most of the chronic non-communicable diseases including diabetes and DN, share the same pathology, the association of DN and DM is complex and bidirectional.

DM is associated with increasing DN (multi-infarct, Alzheimer disease, or diabetes-related), also, Sirtuins, accumulation of advanced glycation end products, and amyloid-β precursor protein are suggested to mediate the cross-talk between DM and neurodegenerative conditions including Alzheimer's (or Alzheimer) disease (AD) [5,6]. Thus, the bridge linking between diabetes and AD is from signaling to all stages of holistic patient care including adherence to lifestyles and medications. Adherence to diabetic medications although integral to patients' well-being. However, the literature lack worldwide. Therefore, the current review aimed to assess the effects of DN effects on adherence to anti-diabetic medications.

## Materials and methods

Eligibility criteria

Studies were included if they were conducted on adult humans suffering from DM and DN and published in the English language. Retrospective, prospective cohorts, case-control, and cross-sectional studies were included, the studies must compare the effects of cognitive impairment on medication adherence or cognitive dysfunction among non-adherent patients. In the present survey, case reports and studies conducted on children were excluded, as were animals and experimental studies.

The outcome measures

The outcome measures were medication adherence to diabetic medications among patients with DN or the association of DN with compliance to medications. We did not specify any type of DN due to the poverty of literature on this important topic. Thus, AD, vascular DN, and diabetes-related DN were included [7].

The search strategy

**Figure 1:**
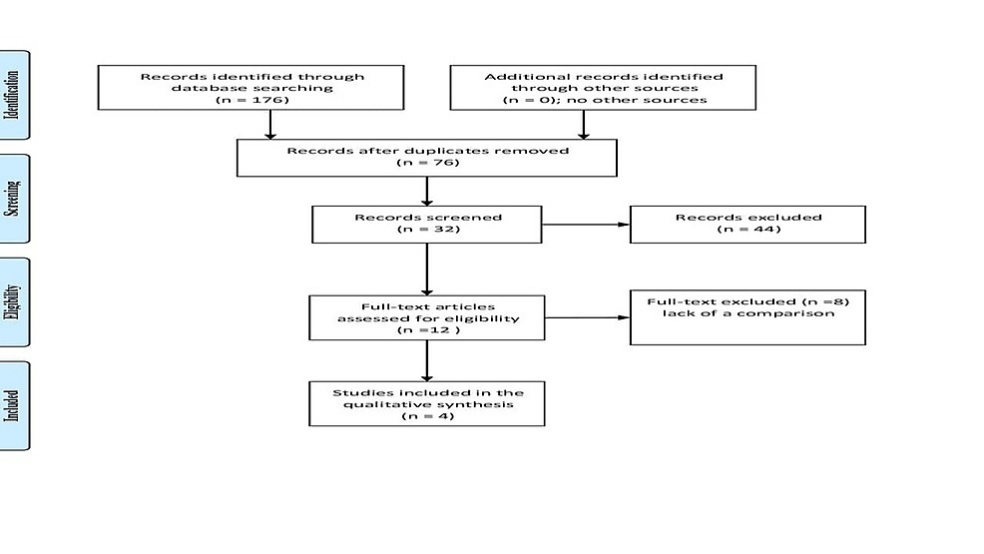
The different phases of the systematic review regarding the association of dementia and adherence to anti-diabetic drugs (the PRISMA chart). PRISMA: Preferred Reporting Items for Systematic Reviews and Meta-Analyses.

We systematically searched the literature through PubMed, Medline, Cochrane library, and the first 100 articles published in Google Scholar and accordance with Preferred Reporting Items for Systematic Reviews and Meta-Analyses guidelines. We included articles publishes in English and conducted on humans, no limitation was set to the date of publication, all the articles were approached from the first published up to March 15, 2021. Two investigators (H. M and S. A.) independently screened the titles and abstracts for relevant articles. An additional search was conducted in the cited articles of the full texts retrieved. The keywords used were Dementia, cognitive impairment, cognitive decline, cognitive dysfunction, diabetes self-care, compliance to anti-diabetic drugs, and medication adherence. Any discrepancy between the authors is to be solved by an agreement. One hundred-seventy-six were identified (58, 18, and 100 in PubMed, Cochrane Library, and Google Scholar, respectively), among the twelve full texts screened, only four fulfilled the inclusion and exclusion criteria. A data extraction sheet was used to extract the author's name, country of publication, publication date, type of studies, number of patients included, and the conclusions. The Ottawa Newcastle scale was used for the assessment of included studies (Table 1, Figure 1). 

**Table 1:** Ottawa Newcastle Assessment for the included studies.

Author	Selection	Compatibility	Outcome	Score
Caballero et al. 2018 [8]	3	2	4	9
Jacob et al. 2018 [9]	3	2	4	9
Li et al. 2017 [10]	3	2	4	9
Mendes et al. 2019 [11]	3	2	4	9

Statistical analysis

We used the most recent RevMan (Cochrane tool, version 5.4). The references were entered manually and the data were entered as dichotomous. The random effect measure was used due to the considerable heterogeneity that was assessed by the Chi-Square and standard difference. A P-value of <0.05 was considered significant. 

## Results

**Figure 2:**
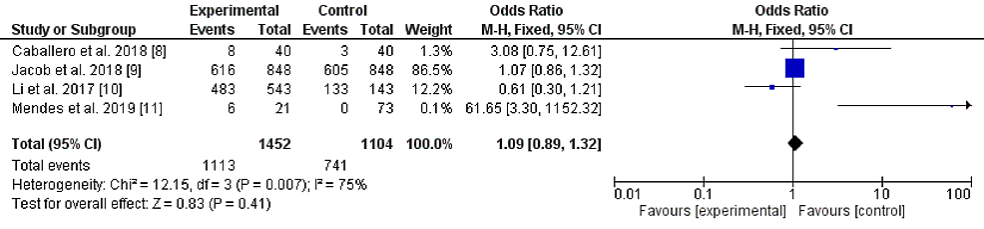
The relationship of dementia and adherence to anti-diabetic medications.

The literature search revealed 176 articles, of the twelve full texts, were screened, and four studies from Europe (two studies, one case-control, and a cross-sectional study), the United States (one cross-sectional study), and Asia (another one study, retrospective) were pooled to assess the relationship of dementia effects on medication adherence [8-11]. The included studies were of good quality (all scored 9 as per Ottawa Newcastle Scale, Table 1). The results showed no effects of dementia on medication adherence, P-value of 0.41, odd ratio: 1.09, 95% CI: 0.89-1.32, Chi-square for heterogeneity: 12.15, I^2^ = 75%, and standard difference = 3. The P-value for heterogeneity was 0.007. The studies included 2,556 patients and 1,854 events. Mendes et al. mostly contributed to the substantial heterogeneity (Table 2, Figure 2).

**Table 2:** The relationship between cognitive impairment and medication adherence.

Author	Year	Country	Type of study	Interventional vs. controls	Significance
Caballero et al. [8]	2018	USA	Older Hispanic patients (cross-sectional)	8/40 vs. 3/40	Significant association
Jacob et al. [9]	2018	Germany	Case-control investigated oral hypoglycemic drugs.	616/848 vs. 605/848	Non-significant
Li et al. [10]	2017	Taiwan	Retrospective carried on elderly community-dwelling	483/534 vs.133/ 143	Non-significant
Mendes et al. [11]	2019	Portugal	Cross-sectional, assessed elderly Hispanic patients.	6/21vs. 0/73	Significant

## Discussion

Cognitive impairment (dementia and mild cognitive dysfunction) is increasingly recognized as a complication of DM among the aging population, recent guidelines recommend screening and optimization of glycemic targets to avoid hypoglycemia and improve medication adherence [12]. The prevalence of DM among elderly patients is alarming (18.8% according to the 2017 estimates, 122.8 million). Importantly, this number is expected to double in the next 30 years. A similar trend was observed for dementia (6%-7%, 46.8 million people in the year 2015, and the number is projected to double by the year 2035) [13,14]. DM, DN, and due to the shared pathology or at least the risk factors usually co-exist and exacerbate each other deleterious consequences, in the United States the combined occurrence of these two morbid disorders is, 13.1% and 24.2% in age groups 65-74 years and ≥75 years, respectively [15].

In the current meta-analysis, no association was found between cognitive decline and adherence to anti-diabetic medications (odds ratio: 1.09, 95% CI: 0.89-1.32). A position statement in the year 2019 emphasized the need for cooperation between the involved healthcare providers (primary healthcare physicians, dialectologists, Psychiatrists, and Geriatricians) to prevent DM and its complications among these at-risk patients [16]. The current findings only mud the water for a big and complex area in diabetes care, the effects of dementia on persistence to diabetes medications although important, but it is not certainly the whole picture. Diabetes holistic care needs to target the different aspects in a wide range of patients from different walks of life and ethnicities and vascular risk factors. There is an interesting notion that thousands of DM exist). Mapping why specific groups of diabetes follow a specific track of complications (poor glycemic control, more severe hypoglycemia, more insulin requirement, and younger or late age of presentation) is vital to provide the specific needs for a special subgroup of diabetes based on the genomic and clinical characters [17]. Impaired insulin signaling and inflammation are shared pathologies in both DM and AD highlighting the importance of Geriatricians in diabetes care in which cognitive function and compliance with the medications are essential components.

The study was limited by the small number of the included studies that were from limited areas and cannot be generalized to the whole world, the limitation to the English language, and the unlimited searching engine time. 

## Conclusions

The current meta-analysis showed no association of cognitive dysfunction and persistence to antidiabetic medications. The small number of the studies investigated and the fact that we targeted both mild cognitive decline and established dementia might be plausible explanations. The considerable heterogeneity among the included studies limited the conclusion. Also, other confounders including family support were not controlled for. Further prospective studies are needed to better inform the diabetes community regarding this important aspect of diabetes care.
